# Structural Analysis of Inhibitor Binding to the Feline Enteric Coronavirus (FECV) Main Protease

**DOI:** 10.3390/v17111506

**Published:** 2025-11-16

**Authors:** Arooma Maryam, Stephanie A. Moquin, Dustin Dovala, Jagroop Kaur, Nese Kurt Yilmaz, Ala M. Shaqra, Celia A. Schiffer

**Affiliations:** 1 Department of Biochemistry and Molecular Biotechnology, University of Massachusetts Chan Medical School, Worcester, MA 01605, USA; 2 Biomedical Research, Novartis, Emeryville, CA 94608, USA

**Keywords:** crystal structure, protease inhibitor, coronaviruses, main protease, drug design

## Abstract

Coronaviruses include various strains that reside in natural animal reservoirs, with zoonotic transmission posing risks to both domesticated animals and human health. Recent efforts to address coronavirus infections have focused on developing inhibitors targeting the main protease (M^pro^), some of which exhibit potential broad-spectrum efficacy. This study presents crystal structures of four clinically relevant inhibitors—GC376, PF-00835231, nirmatrelvir, and ibuzatrelvir—bound to M^pro^ from the feline coronavirus strain FECV-UU23. Structural analysis identified distinct FECV-specific features within the active site where these inhibitors bind and revealed S4 loop as a susceptible structural region essential for the enhanced binding of inhibitors in UU23 M^pro^. We therefore propose to incorporate sterically constrained, functionally tailored heterocyclic moieties at the P3 site of known inhibitors which can optimally engage Q187, P188, and S189 residues of the S4 loop. The findings presented enhance understanding of inhibitor specificity and reinforce the promise of these inhibitor scaffolds for developing antivirals against feline coronavirus strains, with possible applications in broad-spectrum coronavirus therapy.

## 1. Introduction

Coronaviruses pose a significant threat to global health, epitomized by the severe acute respiratory syndrome coronavirus 2 (SARS-CoV-2) which caused the coronavirus disease 2019 (COVID-19) pandemic [1]. The Coronaviridae family comprises diverse enveloped, single-stranded, positive-sense RNA viruses with a striking intrinsic capacity for evolution, adaptation, and diversification. These viruses exhibit varying pathogenic potential, ranging from milder endemic strains to highly pathogenic variants such as SARS-CoV-1 and Middle East respiratory syndrome related coronavirus (MERS-CoV). The pathogenic coronavirus strains likely originated in wildlife (bat) reservoirs, then were transmitted to humans through intermediate mammalian hosts (palm civets, camels) [2]. Cross-species host switching is facilitated by viral mutations and recombination that yield genetically diverse coronavirus strains with modified pathogenicity, altered tissue tropism, and enhanced zoonotic potential [3]. This evolutionary plasticity of the coronavirus family necessitates the investigation of coronavirus diversity, especially across mammalian hosts, to assess their zoonotic transmission potential and pandemic risks. Such efforts are critical to better prepare for future outbreaks and to develop broad-spectrum therapeutic solutions.

Among the broad range of mammalian hosts, domestic cats (felines) have been identified as permissive hosts for diverse coronaviruses, including strains of both porcine and human origin. This vulnerability is specifically concerning in the context of feline coronaviruses, which are in the alpha genus, along with other strains that can infect humans [4]. The zoonotic transmission potential of feline coronaviruses has been evidenced by the detection of a canine–feline related recombinant strain in humans with pulmonary infection [5]. Moreover, reverse zoonosis, particularly human-to-cat transmission of SARS-CoV-2, has also been reported [6]. In the United States, with over 94 million domestic cats, the opportunity for cross-species transmission is significant [7]. Such bidirectional transmission positions felines as potential intermediate hosts for coronavirus evolution, which can facilitate novel variants [8].

The feline coronaviruses are classified into two distinct pathogenic biotypes: the nonvirulent and highly prevalent feline enteric coronavirus (FECV) and the lethal feline infectious peritonitis virus (FIPV). The FECV predominantly infects enteric epithelial cells and causes mild and transient enteritis in domestic cats [4,9]. The FECV-UU23 strain, structurally characterized in the current study, is one of the 11 fully sequenced FECV strains reported so far. In nearly 5% of reported cases, FECV strains including UU23 acquired mutations either in the spike gene or the accessory genes (3abc and of 7ab), enabling efficient replication in feline peritoneal immune cells (macrophages, plasma cells, neurocytes) [10,11]. This mutation-led tropism switching, together with inadequate clearance by immune cells, promoted the evolution of FECV to its highly virulent FIPV variant, characterized by abdominal swelling, blood vessel inflammation, pus formation, and multiple organ failure in cats [12]. In 2023, the severity of this evolved FECV strain was demonstrated in Cyprus, where nearly 300,000 cats, including both stray and domestic, succumbed to death in untreated cases [13].

Following the FIPV outbreak, veterinarians initially relied on RNA-dependent RNA polymerase (RdRP)-targeting antivirals such as GS-441524 and molnupiravir for the treatment of infected cats [14]. This clinical urgency also prompted the structure-guided discovery of GC376, a bisulfite prodrug, as a potent inhibitor against the viral main protease (M^pro^) (PDB ID: 7SNA, 7SMV) [15,16]. Coronaviral M^pro^ is a 3C-like cysteine protease that cleaves the viral polyproteins (pp1a and pp1ab) at evolutionarily conserved sites to release non-structural proteins which are essential for viral replication and transcription in host cells [17]. Due to its indispensable role in viral replication, evolutionary conservation among coronavirus genera (alpha, beta, gamma, delta) (Figure S1), and absence of mammalian homologs, M^pro^ stood out as a promising drug target for developing pan-coronaviral therapeutics. Initially recognized as a veterinary M^pro^ inhibitor, GC376 became a rapid response scaffold for antiviral development against other coronavirus strains. Building on this scaffold, second-generation inhibitors such as PF-00835231 and nirmatrelvir (a component of Paxlovid) were developed [18]. With warhead modifications and optimized pharmacophores at the P2/P3 positions, these compounds retained inhibitory effects across SARS-CoV-2 variants, and we previously characterized them against beta (PDB ID: 8DSU, 8DZ2) and delta (PDB ID: 8E7C) coronaviruses [19,20]. The more recent second-generation standalone oral SARS-CoV-2 inhibitor ibuzatrelvir (PF-07817883) showed cross-reactivity against MERS-CoV M^pro^ [21]. Unlike nirmatrelvir, ibuzatrelvir’s P2 trifluoromethyl proline substitution and P4 methyl carbamate capping group collectively enhance binding affinity to both SARS-CoV-2 and MERS-CoV M^pro^. However, none of these second-generation antiviral candidates have been tested against alpha coronaviruses, including feline variants which demonstrate multi-host adaptability, high recombination potential, and proven zoonotic risks.

In this study, we present crystal structures of M^pro^ from the feline coronavirus strain FECV-UU23 in complex with four clinically relevant inhibitors: GC376, PF-00835231, nirmatrelvir, and ibuzatrelvir. Analyses of these structures revealed FECV-specific features in the active site neighboring the catalytic dyad (Cys144-His41) where the inhibitors bind. The invariant residues together with the structural plasticity of recognition subsites enabled the strain-dependent binding of these broad-spectrum inhibitors to UU23 M^pro^. The findings presented in the current study advance our understanding of inhibitor specificity and support the repurposing of these existing clinical antivirals for veterinary purposes, particularly for FECV infections.

## 2. Materials and Methods

### 2.1. Expression and Purification of Feline Enteric Coronavirus (UU23) M^pro^

The gene encoding UU23 M^pro^ (residues 1–302) was codon optimized, synthesized, and cloned into a pET-based expression plasmid with an N-terminal His-SUMO tag. Plasmid was then transformed into Hi-Control *BL21(DE3) E. coli* cells. A single transformant selected on a kanamycin plate was used to grow overnight Lysogeny Broth (LB) starter culture (with 50 µg/mL kanamycin). This saturated overnight culture was then used to inoculate a Terrific Broth (TB) day culture (1 L), supplemented with 50 mM sodium phosphate (pH 7.0) and 50 µg/mL kanamycin. The day culture was incubated at 37 °C in a shaking incubator (200 rpm) until OD_600_ reached 1–1.5. Protein expression was then induced by adding 0.5 mM Isopropyl ß-D-1-thiogalactopyranoside (IPTG) and temperature was lowered to 19 °C for overnight growth. Cells were then harvested by centrifugation at 6000× *g* for 30 min. For purification, cell pellets were resuspended in lysis buffer (50 mM Tris pH 8.0, 400 mM NaCl, 1 mM TCEP) and lysed through a cell homogenizer. Following lysis, insoluble fraction was isolated from the homogenate by centrifugation at 45,000× *g* for 30 min. The clarified supernatant was then loaded on to a 5 mL Ni-Sepharose Excel column (pre-equilibrated with 5 column volumes of lysis buffer) on an AKTA FPLC at a flow rate of 5 mL/min. The column was washed with lysis buffer until the A_280_ stabilized and bound protein was eluted by modulating the imidazole concentration with a linear gradient over 40 column volumes into elution buffer (50 mM Tris pH 8.0, 400 mM NaCl, 1 mM TCEP, 500 mM imidazole). The fractions obtained from the affinity chromatography were confirmed by SDS-PAGE analysis and UU23 M^pro^ containing fractions were retained only. ESI-LC/MS analysis was further used to confirm the correct molecular weight of His-SUMO-UU23 M^pro^. To cleave the His-SUMO tag, 100 µg of ULP1 was added to the pooled fractions and cleavage was performed at room temperature (RT) overnight during dialysis into 3 L of lysis buffer using a 3500 MWCO dialysis cassette (Thermo Fisher Scientific, Waltham, MA, USA). Although some initial precipitation was observed, protein remained soluble without any further precipitate throughout the rest of the purification and downstream applications. The cleaved protein was passed over 5 mL of Ni-NTA resin pre-equilibrated with lysis buffer to trap His-SUMO tag. The flow through was collected and concentrated to approximately 5 mL for size exclusion chromatography (SEC). Protein was loaded on to a Superdex 75 16/60 column pre-equilibrated with fresh SEC buffer (25 mM HEPES pH 7.5, 150 mM NaCl, 1 mM TCEP) at 1 mL/min flow rate. Fractions from SEC run were analyzed by SDS-PAGE and those containing pure UU23 M^pro^ were pooled, concentrated, aliquoted, and stored at −70 °C. The typical final yield ranged from 40 to 80 mg of pure UU23 M^pro^ per L of bacterial culture.

### 2.2. Co-Crystallization of Feline Enteric Coronavirus (UU23) M^pro^ with Inhibitors

For co-crystallizing UU23 M^pro^ with GC376 (Millipore-Sigma, Burlington, MA, USA), PF-00835231, nirmatrelvir, and ibuzatrelvir (the latter three purchased from MedChemExpress, Monmouth Junction, NJ, USA), 10 mg/mL of UU23 M^pro^ stock was first diluted to 5 mg/mL protein and incubated with 10-fold molar excess (1 mM) of compounds for 1 h at room temperature. Crystals of UU23 M^pro^ with compounds were grown by hanging drop vapor diffusion in pre-greased VDX trays (Hampton Research, Aliso Viejo, CA, USA) at room temperature.

Overnight crystals of UU23 M^pro^ with GC376 and nirmatrelvir were obtained with 10–20% (*w*/*v*) polyethylene glycol (PEG) 3350, 0.1 M Bis-Tris Methane pH 5.5, and 0.2 M NaCl. With PF-0083523, the protein complex crystallized in 0.2 M calcium acetate hydrate, 0.1 M sodium cacodylate trihydrate (pH 6.5), and 40% *v*/*v* PEG 300. For ibuzatrelvir complex, crystals were obtained in H7 (HR2-145-86-JCSG Plus 91) condition, consisting of 0.2 M ammonium sulfate, 0.1 M Bis-Tris pH 5.5, 25% PEG3350. Crystal growth was optimized by testing multiple protein-to-mother liquor ratios: 1 μL:2 μL, 2 μL:2 μL, and 3 μL:2 μL per well, respectively. After a week, diffraction-quality large crystals of UU23 M^pro^ with GC376 and nirmatrelvir were obtained, whereas with ibuzatrelvir crystals grew to beam line quality in two weeks at room temperature. The crystals were cryoprotected using the mother liquor supplemented with 20–25% glycerol prior to data collection. For UU23 M^pro^ crystallization with PF-0083523, crystals were cryoprotected by soaking in cryogenic solutions composed of original precipitant mentioned above. The soaked crystals were mounted on loops and flash-frozen in liquid nitrogen for X-ray diffraction analysis.

### 2.3. Data Collection, Structure Determination, and Analysis

Crystals were sent to Brookhaven National Laboratory NSLS-II Beamline 17-ID-2 (FMX) for data collection at 100 K. At NSLS-II, the collected diffraction intensities were automatically indexed, integrated, and scaled using XDS22. Before the structural analysis, quality assessment of the diffraction data was completed using Xtriage [22]. The reference model used in the current study for molecular replacement was feline coronavirus (FIPV) M^pro^ with GC376 (PDB ID: 7SNA) [16]. Prior to molecular replacement in PHASER [23], all non-protein components including buffer, water, and cryogenic molecules as well as the small molecule inhibitor in the active site of template structure were removed. To avert overfitting (model bias), 95% of the diffraction data was used for structure refinement and R-free was estimated using the 5% excluded data [24].

The MOL2 files of GC376 (PDB: UED), PF-00835231 (PDB: V2M), nirmatrelvir (PDB: 4WI), and ibuzatrelvir (PDB: YDL) were taken from the Protein Data Bank and Gauss view 6 (Gaussian 16) was used for geometry optimization using the basis set: DFT B3LYP 6-311++G (d, p) [25]. With the optimized ligand structures, eLBOW plugin of Phenix created ligand atomic positions, constraint CIF files, and corresponding PDB coordinates necessary to fit the small molecules into their respective densities [26].

Automated iterative model refinement was performed in Phenix (1.20.1–4487), whereas manual model building was performed in Coot (0.9.8.92) [27]. Merohedral twinning was accounted by applying twin law (-h-k, k, -l) while refining structures in Phenix [22]. Comprehensive validation of structures was performed using MolProbity [28] before PDB deposition. Statistics related to X-ray data collection and crystallographic refinement are reported in the Supplementary Table S1.

Before structural analysis, the UU23 M^pro^-inhibitor complexes were first prepared using the Protein Preparation Wizard v.19.2 from Schrödinger Small Molecule Drug Discovery Suite [29]. This procedure involves addition of hydrogen atoms and missing side chains, determination of protonation states, and energy minimization of the structures using OPLS2005 forcefield [29,30]. The missing side chain atoms were added to the protein structures using Prime, and the hydrogen bonds were optimized using PROPKA by sampling the water orientations and assigning protonation state to the residue side chains at pH 7.4 [31]. Finally, the Impref plugin was used for gradient minimization of protein structures (convergence criterion up to 0.5 Å) to fix steric clashes that can occur after the addition of hydrogens or filling missing atoms of protein sidechains. Water molecules were preserved in the structures during energy minimization.

Hydrogen bonds between UU23 M^pro^ active site residues and inhibitors were identified using default angular and distance criteria of PyMol [32]; there was a distance cutoff of 3.6 Å with an angle 63°–180° between donor and acceptor heavy atoms. For each close-contact atom/interacting pair of inhibitors and UU23 M^pro^ complex, van der Waals (vdW) interaction energies were calculated by applying Lennard–Jones potential [33] and summed to obtain the total for each active site residue [34].

### 2.4. Protease Activity and Inhibition Assays

Protease activity of the UU23 M^pro^ was assessed using a fluorogenic substrate, Dabcyl-KTSAVLQSGFRKM-E(EDANS)-NH2, corresponding to M^Pro^ NSP 4/5 cleavage site (GenScript). First, the *K_m_* value of the fluorogenic substrate was determined. Substrate was solvated in 3% Dimethyl sulfoxide (DMSO) and serially diluted from 100 µM stock solution using a buffer (25 mM Bis-Tris, 1 mM DDT (dichloro-diphenyl-trichloroethane) pH 7.0). Using a PerkinElmer EnVision plate reader, 5 µL aliquots of UU23 M^Pro^ were dispensed at a final concentration of 5 nM in each well of a black, polypropylene 96-well half-area plate (Corning, Corning, NY, USA). Fluorescence was measured at 485 nm with excitation at 340 nm. All measurements were taken in duplicate and a 0 µM substrate control was included in each assay. All data were analyzed via Prism (version 9.4.1).

To assay the inhibition of UU23 M^pro^ by GC376, PF-00835231, nirmatrelvir, and ibuzatrelvir, the fluorogenic substrate above was utilized to determine the IC_50_ with Prism (version 9.4.1). A reaction volume of 60 μL was prepared in each well of a black, polypropylene 96-well half-area plate (Corning). At first, inhibitor stocks were dissolved in 4% DMSO and buffer (25 mM Bis-Tris pH 7.0, 1mM DDT) at a working concentration of 550 nM (GC376), 100 nM (PF-00835231), 2.2 µM (nirmatrelvir), and ibuzatrelvir (20 μM). Each inhibitor was serially diluted 2-fold (1/2) in 10 steps. The concentration range of GC376, PF-00835231, nirmatrelvir, and ibuzatrelvir was 250 nM–250 pM, 50 nM–50 pM, 1 μM–1 nM, and 10 μM–10 nM, respectively. Inhibitors were incubated with 5 nM of protease for 60 min prior to the addition of the fluorogenic substrate. Substrate was dispensed using an Envision plate reader (PerkinElmer, Waltham, MA, USA) into each well at a final concentration of 10 µM and fluorescence was measured at 485 nm with excitation at 340 nm. All assays were repeated in triplicate and included a control with no inhibitor. Prism was used to analyze the data (version 9.4.1).

### 2.5. Thermal Shift Assays

Thermal shift assays were performed to assess the impact of inhibitor binding on protein thermostability, using a CFX real-time PCR thermocycler (Bio-Rad, Hercules, CA, USA). Reaction mixtures (50 μL final volume) were prepared in a 96-well Thermo Scientific AB-0700 polymerase chain reaction (PCR) clear plate. Inhibitor stocks dissolved in 100% DMSO were diluted to a final concentration of 50 µM in reaction buffer (25 mM Bis-Tris pH 7.0, supplemented 1 mM DDT), with 5% DMSO. The protein at a final concentration of 5 μM was added to each inhibitor-containing well, followed by incubation for 1 h at room temperature. Control wells included 5 μM of UU23 M^pro^ with 5% DMSO in the absence of inhibitors. After 1 h of room temperature incubation, 5000X SYPRO Orange dye (Thermo Fisher Scientific, Waltham, MA, USA) at a final concentration of 5X in each well was added immediately prior the thermal denaturation. Temperature ramping was performed from 25 to 95 °C, with an increment of 0.3 °C every 12 s. The HEX channel was used to quantify the relative fluorescence units (RFUs) for each well at each temperature interval. Melting temperature (*T_m_*) values were obtained from the maximum value of first derivative (dF/dT) plots of the protein unfolding curves. All assays include triplicate measurements and appropriate no-inhibitor controls.

## 3. Results

**Figure 1:**
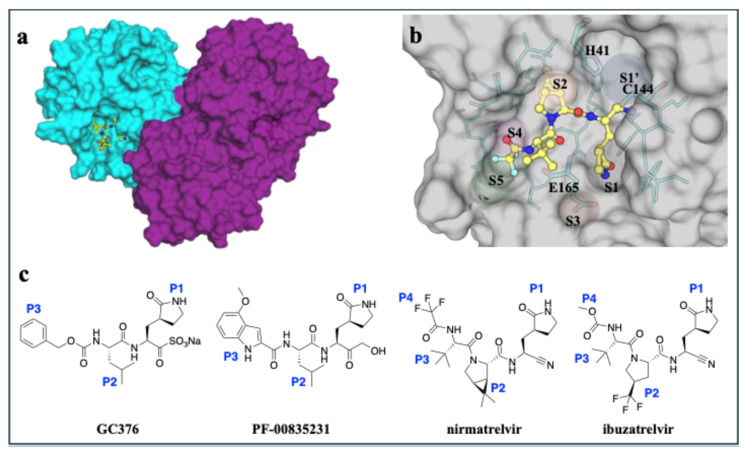
Overview of the structure of M^Pro^ from feline enteric coronavirus (FECV) UU23 bound to inhibitors. (**a**) UU23 M^Pro^ crystal structure in surface representation with the two protomers colored (cyan and magenta) and the inhibitor bound at the active site (nirmatrelvir shown as yellow ball-and-stick). (**b**) Close up view of the active site with key subsites (S1’, S1, S2, S3, S4, and S5) labeled and the active site residues displayed as sticks (cyan with the protein in gray surface representation). (**c**) Chemical structures of GC376, PF-00835231, nirmatrelvir, and ibuzatrelvir with P1, P2, P3, and P4 moieties labeled.

In the current study, we investigated the inhibition of M^pro^ from the UU23 strain with four broad-spectrum protease inhibitors, revealing the structural basis of inhibition in this feline coronavirus variant (Figure 1). To measure the inhibition of UU23 M^pro^ by the compounds, enzymatic inhibition assays were conducted with a fluorogenic substrate (NSP 4/5; *K_m_* = 14 ± 2 µM), as detailed in the Section 2. In the enzyme inhibition assays, all four inhibitors demonstrated nanomolar potency, with PF-00835231 being the most potent (IC_50_ = 3.9 ± 0.2 nM), followed by GC376 (IC_50_ = 12.4 ± 1.3 nM), ibuzatrelvir (IC_50_ = 41.6 ± 5.5 nM), and nirmatrelvir (IC_50_ = 55.7 ± 4.7 nM) against UU23 M^pro^ (Table 1).

Additionally, we used thermal shift assays as an orthogonal method to confirm binding with structural stabilization. The effect of inhibitor binding on protein structure stabilization and thermal stability was probed using differential scanning fluorimetry (DSF). In the absence of inhibitors, apo feline UU23 M^pro^ unfolded at approximately 49.9 °C, while inhibitor bound complexes exhibited a marked increase in thermal stability, as evident from right-shifted melting curves (Table 1 and Figure S2). The binding of GC376, PF-00835231, nirmatrelvir, and ibuzatrelvir to UU23 M^pro^ conferred thermal stabilization, with observed melting temperature (*T_m_*) of 71.4 °C (Δ*T_m_* = 21.5 °C), 70.6 °C (Δ*T_m_* = 20.7 °C), 65.5 °C (Δ*T_m_* = 15.6 °C), and 61.3 °C (Δ*T_m_* = 11.4 °C), respectively (Table 1). The increase of *T_m_* values in this orthogonal assay confirmed binding of all four compounds to UU23 M^pro^.

All four inhibitors were co-crystallized with UU23 M^pro^ to reveal the structural basis of inhibition in this feline coronavirus variant. Co-crystallized structures of UU23 M^pro^ with GC376 (PDB ID: 9MVL) and PF-00835231 (PDB ID: 9MW4) were solved in P 43 2 2 space group as one monomer per asymmetric unit at 1.5 Å resolution. UU23 M^pro^ with nirmatrelvir (PDB ID: 9MVK) and ibuzatrelvir (PDB ID: 9PQG) structures were determined in P 1 21 1 and P 21 21 21 space groups, respectively, each containing four protomers in one asymmetric unit. Diffraction data for these structures were processed at 1.97 Å and 2.27 Å (Table S1). The structures showed an invariant catalytic dyad, as expected, along with structurally conserved subsites despite sequence divergence and active site plasticity, critical to tolerate broad-spectrum inhibitor binding in coronavirus strains.

### 3.1. Crystal Structure of GC376 with Feline UU23 M^pro^

**Figure 2:**
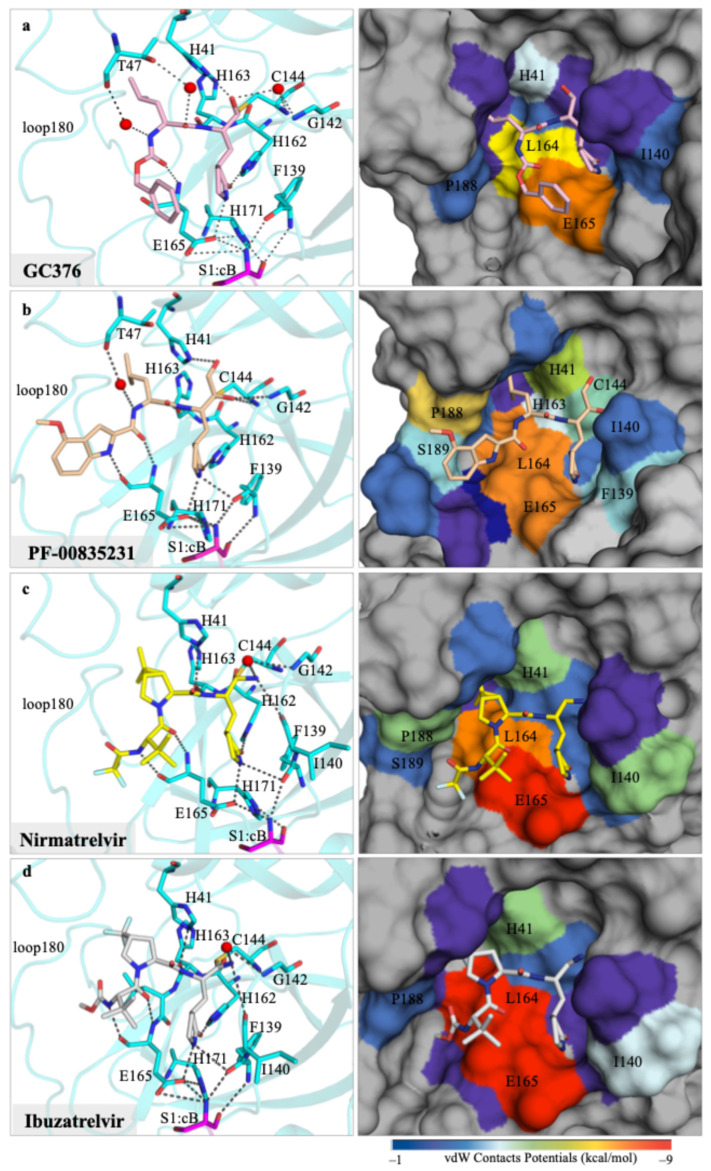
Crystal structures of feline coronavirus UU23 M^pro^ with four inhibitors, focusing on the molecular interactions at the active site. Hydrogen bonding network (black dashed lines) between the inhibitor (ball-and-stick) and protease’s (cyan cartoon representation) active-site residues (cyan sticks) are displayed on the left-hand-side panels for (**a**) GC376, (**b**) PF-00835231, (**c**) nirmatrelvir, and (**d**) ibuzatrelvir. The panels on the right-hand column show the protein in surface representation with the active site residues colored from blue to red for increasing vdW interactions with the bound inhibitor (ball-and-stick).

Analysis of the UU23 M^pro^ cocrystal structure with GC376 revealed covalent bond formation between the aldehyde warhead and catalytic C144, as expected. The P1’ position was further stabilized via water-mediated hydrogen bonds of the backbone oxyanion with H41 and G142 (Figure 2a). The γ-lactam P1 group of GC376 established multiple hydrogen bonds with residues F139, H162, and H163 in the S1 subsite. The flexible leucine-like P2 group was restricted into the S2 subsite mainly through hydrophobic interactions with L164. Notably, in this complex, two water molecules coordinated interactions between GC376 and T47, further stabilizing binding of the P2 group in the S2 subsite (Figure 2a). These water molecules are missing in the SARS-CoV-2 (beta-CoV) structure where the P2 moiety has a slightly altered orientation and the S2 subsite is more hydrophobic and smaller, mainly due to the larger M49 and Q189 (Figure S3).

The P3 benzyl moiety was bound in the UU23 M^pro^ active site in a conformation similar to that in SARS-CoV-2, with conserved hydrogen bonding of the backbone with E165. This interaction together with minimal vdW contribution from the S4/S5 subsites oriented the P3 ring proximal to the γ-lactam (Figure 2a). Structural alignment of UU23 M^pro^ (FECV) and FIPV M^pro^ (PDB ID:7SNA) revealed that despite 11 amino acid substitutions (97% sequence identity) (Figure S4), the two feline M^pro^ variants retained an identical binding mode of GC376. The overall conserved binding mode across these variants is in agreement with the overall broad-spectrum activity of GC376 against coronaviruses.

### 3.2. Crystal Structure of PF-00835231 with Feline UU23 M^pro^

In each protomer of the UU23 M^pro^ crystal structure, PF-00835231 was stabilized by multiple hydrogen bonds and vdW interactions with the active site residues. The warhead (P1’) of PF-00835231 covalently bound to the catalytic C144 and formed a hydrogen bond with G142 in the S1’ subsite (Figure 2b). Similarly to GC376, the S1 subsite engaged the glutamine mimic γ-lactam moiety (P1) through several hydrogen bonds. The indole group (P3 moiety) of PF-00835231 interacted with the backbone atoms of E165 (S3 subsite) through a hydrogen bond. Importantly, the leucine moiety (P2) of the inhibitor was tucked in the S2 subsite with the coordination of a water-mediated hydrogen bond with residue T47. Enhanced vdW interactions with L164, P188, and S189 from the S3/S4 subsites and F139 and L140 from the S1 subsite likely contribute to the tight binding of PF-00835231’s indole and glutamine mimic γ-lactam moiety (Figure 2b).

Structural comparison of PF-00835231 binding in UU23 M^pro^ with previously reported structures of beta and delta coronaviruses [19] reveal that the loops surrounding the inhibitor show distinct conformations where the active sites interact with the inhibitor in a variant-specific manner (Figure S5). Beta and delta coronavirus M^pro^ amino acid sequences share 44% and 36% identity with UU23 M^pro^, respectively (Figure S1). Despite variations in loop conformation and sequence, the binding mode of PF-00835231 in the active pocket is highly conserved (RMSD < 0.2 Å) (Figure S5a,b). The key residues interacting with PF-00835231 (H41, C144/C145, F139/F140, H162/H163, H163/H164, E165/E166) are highly conserved across M^pro^ variants (Figure S1). The primary distinction among the variants is the size and openness of the active site. In beta and delta M^pro^, E188/Q189 from the S3 subsite and the side chain of N141/N142 from the S1’ subsite interact over the inhibitor through hydrogen bonding. This results in the encapsulation of the inhibitor and decreases solvent exposure in delta and beta M^pro^ variants (Figure S5c–e). As a result, the warhead (P1’) and γ-lactam moiety (P1) of PF-00835231 were completely buried in beta and delta coronavirus complexes, whereas in UU23 M^pro^, the wider active site resulted in the inhibitor being more solvent-exposed. Additionally, the sequence variation in the active site pockets, specifically in the S1’ (residue position 140–145), S2 (loop40), and S3/S4 (loop180) subsites, correlated with subtle variations in the inhibitor binding mode and the conformation of the active site loops (Figure S5f–h).

Despite these subtle variations, the overall binding mode and key interactions with the active site are maintained in all complexes with an extensive network of hydrogen bonds, which likely facilitates the binding of PF-00835231 in UU23 M^pro^ similar to other coronaviruses.

### 3.3. Crystal Structure of Nirmatrelvir with Feline UU23 M^pro^

The binding pose of nirmatrelvir in the UU23 M^pro^ is highly similar to that in SARS-CoV-2 M^pro^ (Figure S6). An essential conserved hydrogen bond network with the S1’ subsite (G142, C144), S2 subsite (F139, H162, H163), S3 subsite (E165), and a water-mediated hydrogen bond with backbone atoms of L140 anchor nirmatrelvir in the UU23 M^pro^ active site (Figure 2c). Unlike GC376 and PF-00835231, nirmatrelvir has a large and more rigid bicyclic P2 moiety (Figure 1), which replaced the water-mediated hydrogen bond between the inhibitor and residue T47 present in the other two cocrystal structures (Figure 2a,b). Despite the loss of the hydrogen bond donor atom at the P2/P3 amide linkage in nirmatrelvir, the 6,6-dimethyl-3-azabicyclo [3.1.0] hexane P2 moiety was able to make extensive vdW interactions in the S2 subsite and the side chain of L164 (S3 subsite) of UU23 M^pro^ (Figure 2c).

The comparison of UU23 (alpha) and SARS-CoV-2 (beta) M^pro^ variants revealed variations in the nirmatrelvir binding mode and conformation of the flexible loops at the active site (Figure S6). The inhibitor was shifted away from the 180s loop and the P1’ moiety was closer to the loop residues L140 and N141 (S1 and S1’ subsites) compared to SARS-CoV-2 M^pro^. Additionally, the inhibitor was more solvent exposed as the interaction of M49 with the conserved catalytic residue H41 that locks the P2 group in the S2 subsite was missing in UU23. Substitutions, particularly of M49, N141, and Q189 that shape the active site in SARS-CoV-2 M^pro^, resulted in variations in the loop conformations including the way active pocket contours around the γ-lactam moiety (P1) in SARS-CoV-2 versus UU23 M^pro^ (Figure S6b–e). Additionally, the inhibitor’s rigid bicyclic P2 group may hinder subtle rearrangements to adapt to active site variations, which may cause a shift in the overall binding mode and reduced potency against UU23 M^pro^.

### 3.4. Crystal Structure of Ibuzatrelvir with Feline UU23 M^pro^

The most recent clinical M^pro^ inhibitor ibuzatrelvir is an analog of nirmatrelvir with the bicyclic P2 moiety replaced with a more flexible ring, which we co-crystallized with UU23 M^pro^. As with nirmatrelvir and other irreversible covalent inhibitors, ibuzatrelvir’s nitrile warhead bound covalently to the C144 residue (thiol group) of UU23 M^pro^. The conserved P1 lactam ring of the inhibitor established the same hydrogen bond network in the S1 pocket, including with the side chain of E165 and backbone carbonyl of F139 (Figure 2d). The distinct feature of ibuzatrelvir is the trifluoromethyl proline derivative at the P2 position (Figure 1), which made extensive hydrophobic vdW interactions with H41, D186, Q187, and P188. The tert-butyl P3 moiety was able to establish hydrogen bonds and extensive vdW interactions with E165 and L164. The CH_3_ moiety of P4 methyl carbamate in ibuzatrelvir was sterically accommodated in the pocket by the proximal residues L166, P188, S189, and Q191 (≤−1.5 kcal/mol) that shape the S4 pocket. This arrangement optimally positioned the P4 moiety within the S4 subsite, contributing to the overall stability of the inhibitor–protease complex. Despite the conserved hydrogen bond network and extensive vdW interactions (−135 kcal/mol), ibuzatrelvir overall was similar to nirmatrelvir in inhibiting UU23 M^pro^.

The structural comparison of ibuzatrelvir bound to UU23 versus SARS-CoV-2 M^pro^ indicated subtle conformational rearrangements in inhibitor binding (Figure S7). The active site of UU23 M^pro^ in the presence of ibuzatrelvir exhibited a more open conformation overall due to the outward movement of loop180 and minimal interloop contact between loop180 and loop40 compared to SARS-CoV-2, where ibuzatrelvir was more buried (Figure S7b,c). Sequence variation between the two protease variants in key subsite recognition regions resulted in certain conformational changes (Figure S7d,e), but the hydrogen bond networks involving the catalytic residues were conserved (Figure 2d). The weakened affinity of ibuzatrelvir to M^pro^ from UU23 versus SARS-CoV-2 (reported < 20 nM [21]) might be due to the less favorable hydrophobicity (S2 subsite) and complementarity (S4 subsite).

## 4. Discussion

The COVID-19 pandemic has established the lethal potential of coronaviruses, as exemplified by SARS-CoV-2, a highly transmissible infectious agent capable of infecting a broad range of mammalian hosts. The diversity of coronaviruses circulating in the animal population and the risk of recurring coronavirus spillovers at the animal–human interface necessitate preemptive research on divergent strains to block plausible future pandemics. Domestic cats represent a particularly concerning conduit in cross-species transmission by being both natural coronavirus carriers and susceptible hosts of SARS-CoV-2. The intersection of veterinary and human public health risks mandates structural characterization of the viral proteins to facilitate rational design and development of cross-reactive antivirals for pandemic preparedness. As part of our research to study divergent mammalian-infecting coronaviruses [19,35], we determined high-resolution crystal structures of M^pro^ from the UU23 strain of feline alpha coronaviruses with four potentially broad-spectrum inhibitors: GC376, PF-00835231, nirmatrelvir, and ibuzatrelvir.

Our structural findings reveal that the active site of UU23 M^pro^ maintained conserved catalytic residues in addition to distinct, lineage-specific structural plasticity, especially in the loops forming the substrate binding subsites. Cocrystal structures with the four inhibitors presented in the current study indicate that UU23 M^pro^ had altered the conformation of loop40 (S2 subsite) and loop180 (S4 subsite), distinct from SARS-CoV-2 and other related main proteases. In the crystal structure we solved previously of M^pro^ from a delta coronavirus, the salt bridge between K45 (loop40) and E188 (loop180) brings S2 and S4 subsites close to the bound inhibitor and S1 region [19]. Similarly, in SARS-CoV-2, a polar network involving R40, M49, and L50 from loop40 with D187 and R188 from loop180 helps the S2/S4 cleft to partially enclose the inhibitor [18,19]. UU23 M^pro^ lacks these key interactions and substitutions in loop40 (V50, I51) and loop180 (Q187) changed the active site configuration at the S2/S4 subsites. Unlike beta and delta, UU23 M^pro^ has a more open active site. Nevertheless, inhibitor binding was maintained through conserved catalytic residues and compensatory conformational adjustments.

Our results confirm the lower nanomolar potency of GC376 (IC_50_ ~12.4 ±  1.3 nM) against the FECV variant, reaffirming its established efficacy against FIPV (IC_50_ ~130  ±  20 nM) and SARS-CoV-2 (IC_50_ ~26.4  ±  1.1 nM, 190  ±  20 nM) as well as broad-spectrum inhibition of 3C and 3C-like proteases including M^pro^ [16,35,36,37]. We also evaluated the potency of PF-00835231, nirmatrelvir, and ibuzatrelvir against the FECV variant where PF-00835231 outperformed the latter two. This finding is consistent with PF-00835231’s well characterized inhibition against SARS-CoV-2 M^pro^ (IC_50_ ~0.27 nM–8 nM) and other alpha, beta, and gamma variants (*Ki* = 30 pM and 4 nM) [38]. The hydrophilic S2 subsite of UU23 M^pro^ nicely accommodates the less bulky P2 of GC376 and PF-0083523 through water-mediated interactions as compared to the nirmatrelvir and ibuzatrelvir’s substantially larger P2 substituents. Additionally, the polar S3 pocket (E165) together with the moderately hydrophobic yet flexible S4 region of UU23 M^pro^ accommodate the polar P3 indole moiety of PF-00835231 better as compared to the flexible P4 moieties of nirmatrelvir and ibuzatrelvir.

This induced fit binding agrees with the reduced potency of the latter two inhibitors against UU23 M^pro^ (Table 1) compared to SARS-CoV-2 variants. Substitution of key residues in UU23 M^pro^ altered the arrangement of loops that moderately increased the nirmatrelvir’s potency, elevating its IC_50_ ~7.9 nM–10.5 nM (against SARS-CoV-2 variants including omicron and delta) to 55.7 ± 4.7 nM. Ibuzatrelvir, likewise, showed reduced potency against UU23 M^pro^, with an IC_50_ of 41.6 ± 5.5 nM compared to 19 nM ± 4 nM against SARS-CoV-2 [21,31].

To improve the potency of the inhibitors against the feline variants, the stabilization of S4 (loop180) could be achieved by adding more polar and sterically constrained substituents that can optimally engage the Q187, P188, and S189 residues of the S4/S5 of UU23 M^Pro^. In addition to providing insights into drug design, our structural analysis indicates that the inhibitor scaffolds investigated are promising to develop antivirals to target feline coronavirus strains and potentially achieve broad-spectrum coronavirus therapeutics.

## Figures and Tables

**Table 1:** Inhibitory potency (IC_50_; mean and standard error from three replicates) and thermal stability (*T_m_*, Δ*T_m_*) of feline UU23 M^pro^ with GC376, PF-00835231, nirmatrelvir, and ibuzatrelvir.

Inhibitor	IC_50_ (nM)	*T_m_* (°C)	Δ*T_m_* (°C) *
apo (none)	-	49.9	-
GC376	12.4 ± 1.3	71.4	21.5
PF-00835231	3.9 ± 0.2	70.6	20.7
nirmatrelvir	55.7 ± 4.7	65.5	15.6
ibuzatrelvir	41.6 ± 5.5	61.3	11.4

* Δ*T_m_* was calculated relative to apo UU23 M^pro^. Lower IC_50_ indicates stronger inhibition.

## Data Availability

The data for the crystal structures presented in this study are available in the Protein Data Bank with accession numbers 9MVL, 9MW4, 9MVK, and 9PQG.

## Supplementary Materials

The following supporting information can be downloaded at https://www.mdpi.com/article/10.3390/v17111506/s1, Figure S1: Amino acid sequence alignment for M^pro^ from coronavirus genera; Figure S2: Melting curves showing increase in thermostability of UU23 M^pro^ in the presence of inhibitors; Figure S3: Structural comparison of GC376 binding to alpha and beta coronavirus M^pro^; Figure S4: Sequence and structural comparison of feline M^pro^ variants; Figure S5: Comparative structural analysis of PF-00835231 binding to M^pro^ from alpha, beta, and coronaviruses; Figure S6: Structural analysis of nirmatrelvir binding to M^pro^ from alpha and beta coronavirus variants; Figure S7: Structural analysis of ibuzatrelvir binding to M^pro^ from alpha and beta coronavirus variants; Table S1: Crystallographic X-ray data collection and structure refinement statistics for UU23 M^pro^ in complex with inhibitors.

## Abbreviations

The following abbreviations are used in this manuscript:

M^pro^	Main protease
SARS-CoV-2	Severe acute respiratory syndrome coronavirus 2
MERS-CoV	Middle East respiratory syndrome related coronavirus
FECV	Feline enteric coronavirus
FIPV	Feline infectious peritonitis virus
NSP	Nonstructural protein
IC_50_	Half maximal inhibitory concentration
DSF	Differential scanning fluorimetry
vdW	van der Waals
