# Supplementary material for: Structural Analysis of Inhibitor Binding to the Feline Enteric Coronavirus (FECV) Main Protease
Source: Viruses. 2025 Nov 16;17(11):1506. doi: 10.3390/v17111506 (PMC12656910; doi:10.3390/v17111506)
Supplement: Supplementary file 1 [file viruses-17-01506-s001.zip › viruses-3904506-supplementary.pdf]

Supplementary Information

Structural Analysis of Inhibitor Binding to the Feline Enteric Coronavirus (FECV) Main Protease

Arooma Maryam [1], Stephanie A. Moquin [2], Dustin Dovala [2], Jagroop Kaur [1], Nese Kurt Yilmaz [1], Ala M. Shaqra [1,*] and Celia A. Schiffer [1,*]

[1] Department of Biochemistry and Molecular Biotechnology, University of Massachusetts Chan Medical School, Worcester, MA 01605, USA
[2] Biomedical Research, Novartis, Emeryville, CA 94608, USA
* Correspondence: ala.shaqra@umassmed.edu (A.M.S.); celia.schiffer@umassmed.edu (C.A.S.); Tel.: +1-(508)-856-8008 (C.A.S.)

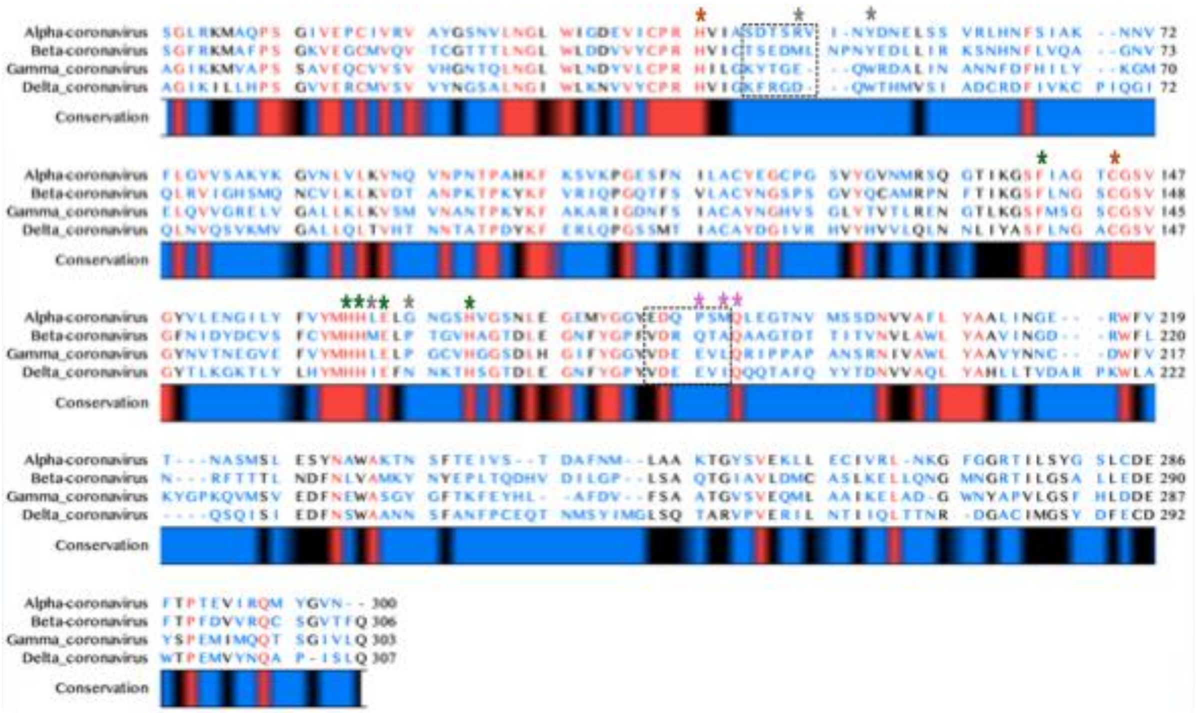

Figure S1. Multiple sequence alignment of alpha-coronavirus (feline enteric coronavirus strain UU23) main protease (M^{pro}) with other representative strains of coronaviral genera: beta-coronavirus (SARS-CoV2), gamma-coronavirus (beluga whale coronavirus SW1), and delta-coronavirus (porcine coronavirus HKU15). Amino acid sequence conservation is indicated in a blue-to-red gradient; with fully conserved residues (100% identity) colored red, moderately conserved residues (≥75% identity) in black, and variable regions (<75% identity) in blue. The essential and highly conserved catalytic residues of M^{pro} are marked with orange asterisks, and key substrate binding subsites are specified by colored asterisks: S1 (green, conserved), S2 (grey, divergent), and S3/S4 (magenta, divergent). Residues located on loop 40 and loop 180 in each variant are enclosed in a black dotted boxes.

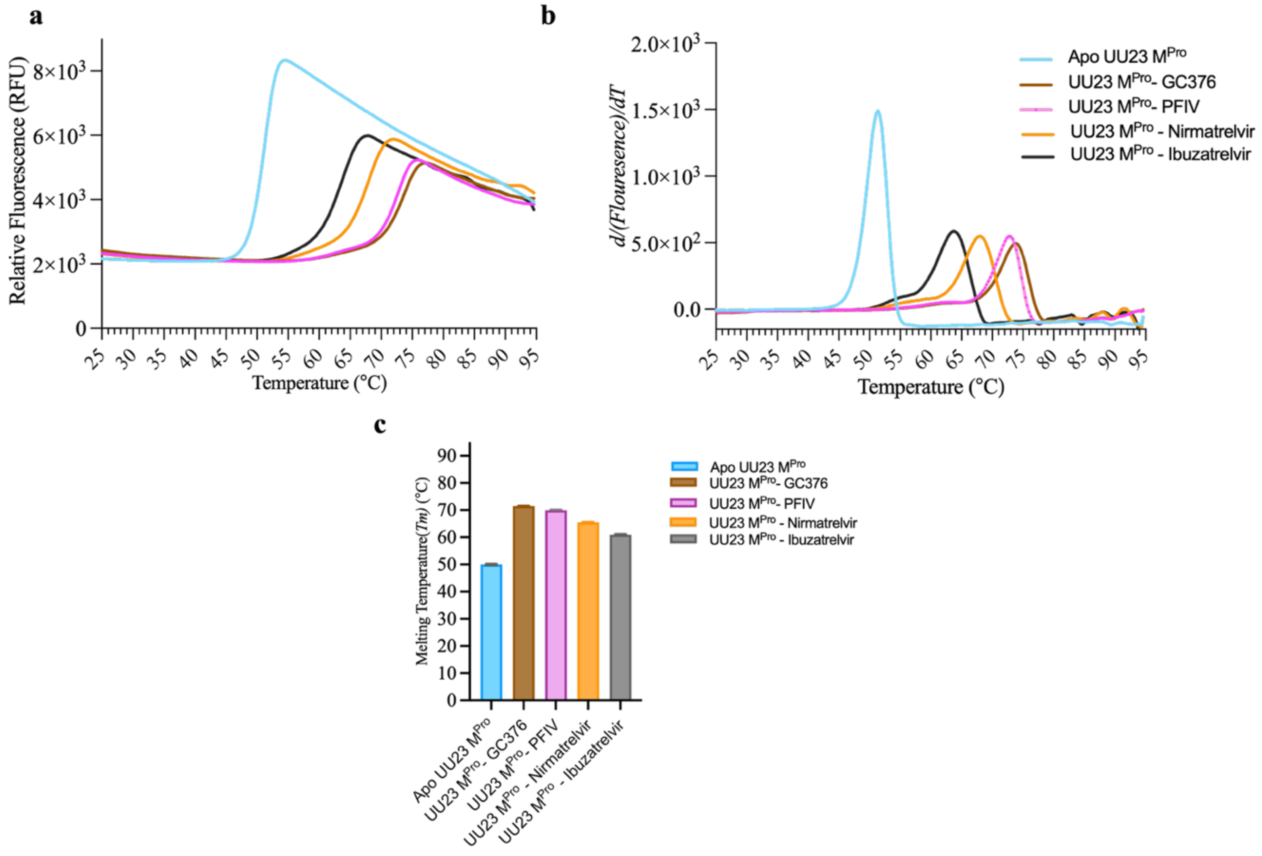

Figure S2. Increase in thermostability of UU23 Mpro with binding of inhibitors. (**a**) Melting curves from DSF measured as fluorescence intensity as a function of temperature for apo UU23 Mpro (cyan) and inhibitor-bound complexes. (**b**) First derivative analysis of DSF melting curves, indicating the rate of fluorescence change (dF/dT) as a function of temperature in the absence and presence of inhibitors. (**c**) The bar graph compares the mean melting temperature (*Tm* in °C) ± standard error mean (SEM) of apo and four inhibitor-bound complexes of UU23 Mpro, based on three replicates (n=3).

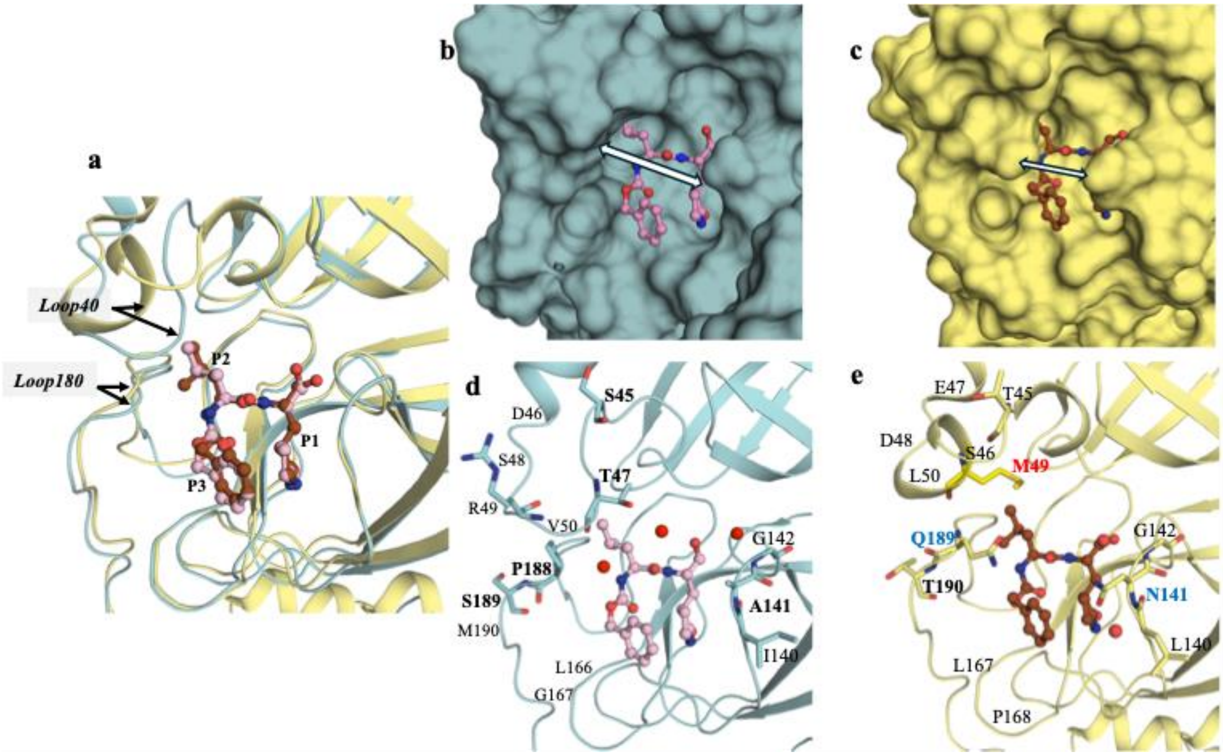

Figure S3. Structural comparison of GC376 binding to alpha and beta coronavirus Mpro. (a) Structural alignment of FECV UU23 (cyan) and SARS-CoV-2 Mpro (PDB ID: 7CBT; yellow) with bound GC376 (pink and brown ball-and-sticks, respectively) demonstrating well-aligned P1 and P2 moieties and shift of the P3 moiety. Arrows point to the residue positions on the key loops (labeled) that change the inhibitor's binding. (b & c) Surface representations of FECV UU23 and SARS-CoV-2 Mpro highlighting genus-specific spatial organization of the active site loops. The binding pocket of UU23 Mpro (cyan) looks more open as compared to the constricted binding pocket of SARS-CoV2 Mpro (yellow). (d & e) Sequence comparison of active site loops, highlighting the variations between the FECV UU23 Mpro and SARS-CoV-2 Mpro. Critical polar residues (labeled in blue) near GC376 in SARS-CoV-2 Mpro that mediate a constricted biding pocket are substituted with non-polar residues in UU23 Mpro. Active site water molecules are shown as red spheres.

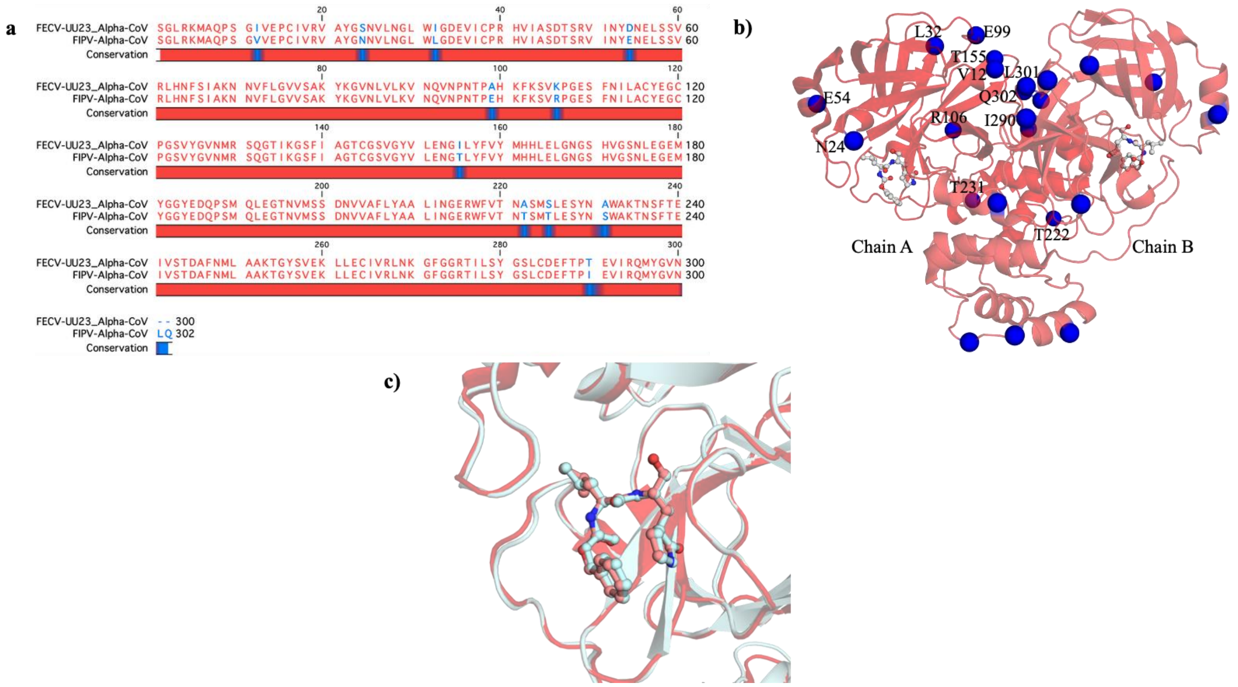

Figure S4. Sequence and structural comparison of feline main protease (M^{pro}) variants. (**a**) Pairwise sequence alignment of M^{pro} from feline enteric coronavirus (FECV: UU23) and feline infectious peritonitis virus (FIPV); both are from the alpha-coronavirus genus. The conserved amino acid residues are colored red while dissimilar residues are shown in blue. (**b**) Substitutions between UU23 (FECV) M^{pro} and FIPV are mapped on the protein structure (blue spheres). (**c**) Comparison of the binding mode of GC376 co-crystallized with M^{pro} of FECV UU23 (light cyan) and FIPV M^{pro} (PDB ID:7SNA; red), demonstrating the conserved inhibitor conformation

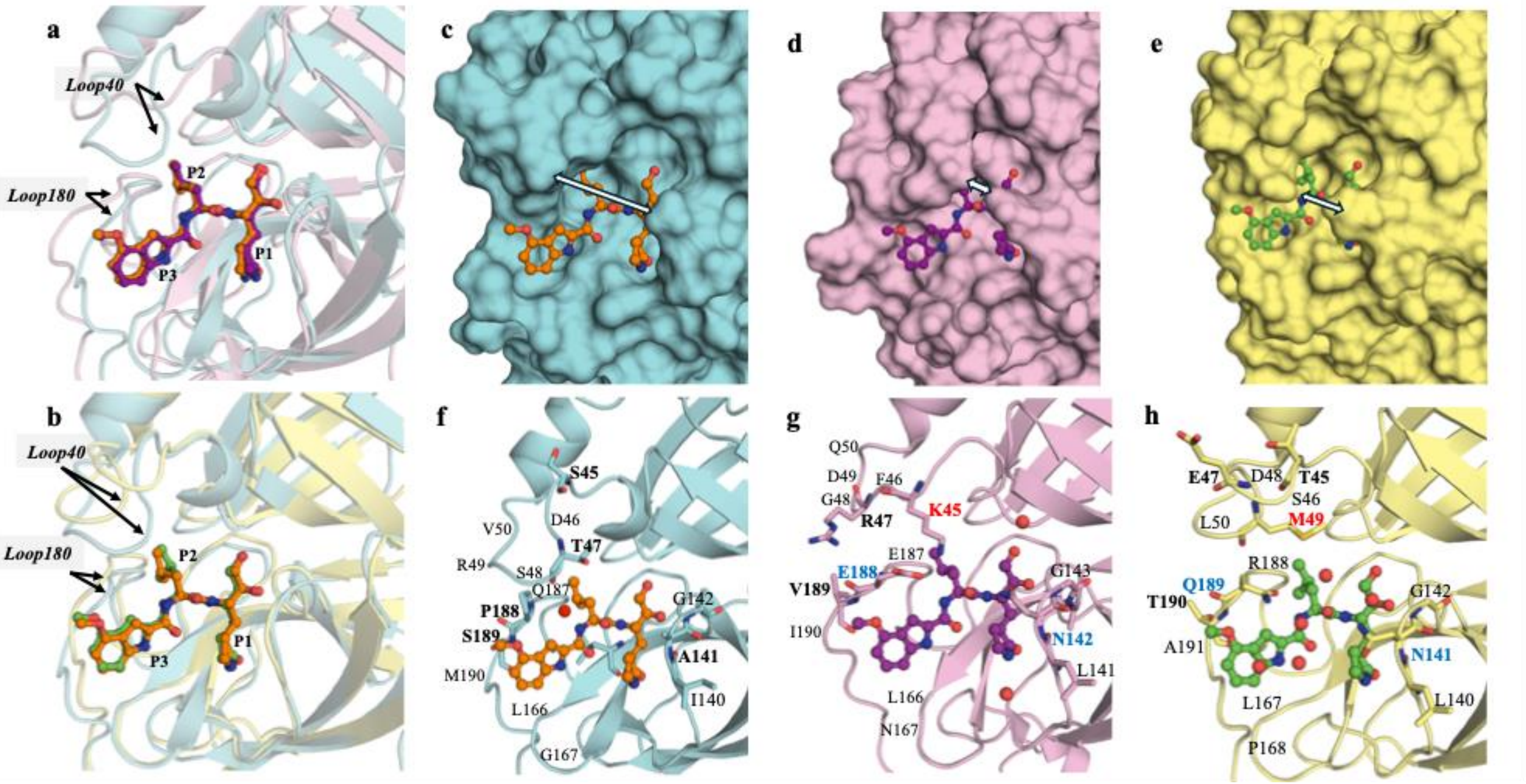

Figure S5. Comparative structural analysis of PF-00835231 binding to M^{pro} from alpha (FECV UU23), beta (SARS-CoV-2) and delta (porcine HKU-15) coronaviruses. (**a** & **b**) Structural alignment of FECV UU23 M^{pro} (PDB Id: 9MW4; cyan), SARS-CoV2 M^{pro} **(**PDB Id: 8DSU; yellow) and delta (PDB Id: 8E7C; pink) demonstrating conserved P1 binding conformation (orange, magenta and green ball and stick, respectively), whereas P2 and P3 substituents showed minor conformational variations across the variants. Arrows point to the residue positions on the key loops (labeled) that change the inhibitor's binding. (**c**, **d**, & **e**) Surface representations of FECV UU23 (alpha), delta and SARS-CoV-2 M^{pro} (beta) highlight variant dependent arrangement of loops that dictates binding pocket contour around the ligand. The binding pockets of delta and beta-CoV effectively encapsulates PF-00835231 compared to alpha CoV. (**f**, **g**, & **h**). Sequence divergence of active site loops across FECV UU23 (alpha), delta and SARS-CoV-2 (beta) M^{pro} bound to PF-00835231. Polar residues labeled in blue interacts directly with inhibitor and stabilize loop folding over it, and residues in red mediate inter-loop salt bridges and VdW contacts to make pocket more compact in delta/beta as compared to alpha variant. Active site water molecules are shown as red spheres.

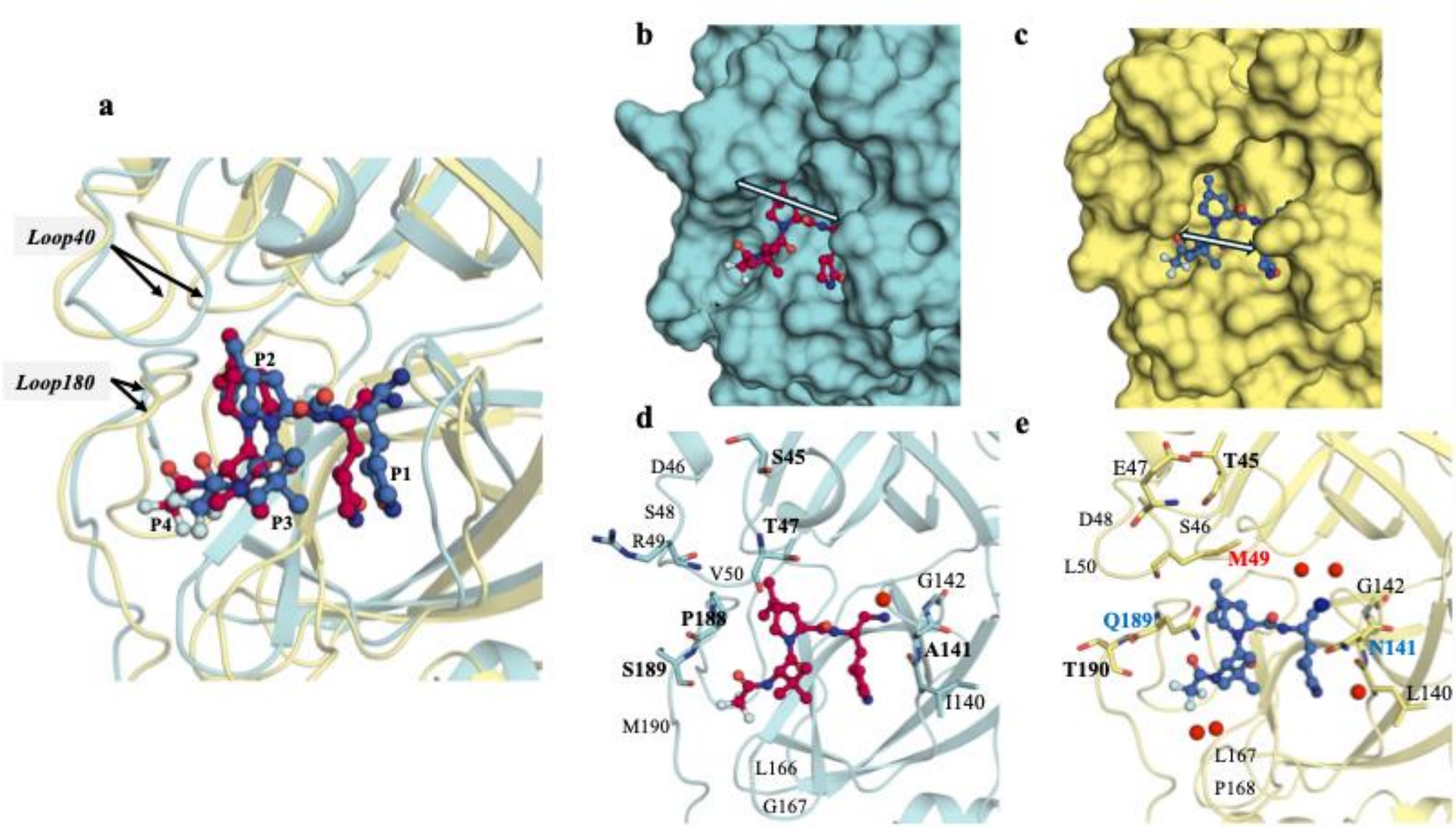

Figure S6. Structural analysis of nirmatrelvir binding to M^{pro} from alpha and beta coronavirus variants. (**a**) Structural superposition of cartoon representations of FECV UU23 (alpha genus; cyan) and SARS-CoV-2 M^{pro} (beta genus; PDB ID: 8DZ2, yellow) highlights distinct arrangement of the active site loops and nirmatrelvir (hot pink and blue ball-and-sticks, respectively). Arrows point to the residue positions on the key loops (labeled) that change inhibitor's binding. Surface representations of (**b**) FECV UU23 and (**c**) SARS-CoV-2 M^{pro} active sites show differential packing of the loops around the inhibitor and a more solvent exposed binding pocket in UU23 M^{pro} as compared to SARS-CoV-2 M^{pro}. Cartoon representations of (**d**) FECV UU23 and (**e**) SARS-CoV-2 M^{pro} with labelled amino acid substitutions (side chains in sticks) that contribute to the structural difference in these inhibitors bound complexes. Active site water molecules are shown as red spheres.

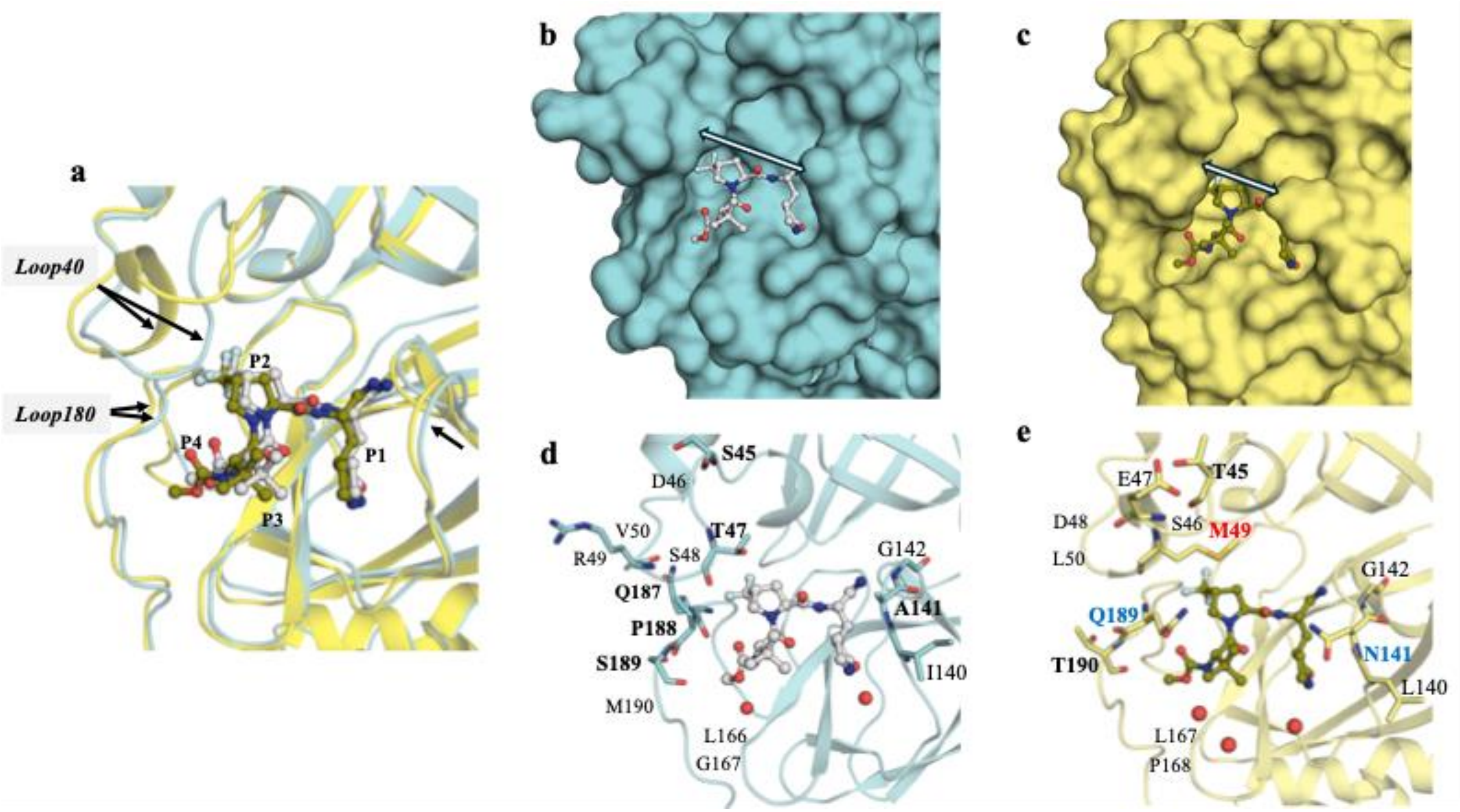

Figure S7. Structural analysis of *i*buzatrelvir binding to M^{pro} from alpha and beta coronavirus variants. **a**) Structural superposition of cartoon representations of FECV UU23 (alpha genus; cyan) and SARS-CoV-2 M^{pro} (beta genus; PDB ID: 8DZ2, yellow) highlights the distinct arrangement of *the* active site loops (labeled) *and i*buzatrelvir (grey and olive ball-and-stick*s*, respectively). Arrows point to the residue positions on the key loops (labeled) that change inhibitor's binding. Surface representations of *(**b**)* FECV UU23 and *(**c**)* SARS-CoV-2 M^{pro} (beta-CoV; yellow) active sites shows differential packing of active site around the inhibitor. In SARS-CoV-2 M^{pro} *the inhibitor's* P1 moiety is partially buried whereas in UU23 M^{pro} *i*buzatrelvir is more solvent exposed. Cartoon representations of (**d**) FECV UU23 and (**e**) SARS-CoV-2 M^{pro} with labelled amino acid substitutions (side chains in sticks) that contribute to the structural difference in these inhibitors bound complexes. Active site water molecules are shown as red spheres.

Table S1. Crystallographic X-ray data collection and structure refinement statistics for UU23 M^{pro} in complex with inhibitors.

**Inhibitor**	**GC376**	**PF-00835231**	**Nirmatrelvir**	**Ibuzatrelvir**
**PDB ID**	9MVL	9MW4	9MVK	9PQG
**DATA COLLECTION**				
Location	NSLS-II	NSLS-II	NSLS-II	NSLS-II
Resolution Range (Å)	34.51-1.50 (1.55-1.50)	34.52-1.50 (1.55-1.50)	33.59-1.97 (2.04-1.97)	34.18-2.27 (2.35-2.27)
Space Group	P 4_3 2 2	P 4_3 2 2	P 1 2_1 1	P 2_1 2_1 2_1
a,b,c, (Å)	77.166, 77.166, 150.685	77.193, 77.193, 149.563	63.919, 106.336, 78.509	80.656, 97.754, 204.563
alpha, beta, gamma (°)	90, 90, 120	90, 90, 90	90, 90.512, 90	90, 90, 90
Total Reflections	147146 (14494)	146124 (14386)	143423 (14719)	150969 (14856)
Unique Reflections	73573 (7247)	73062 (7193)	72222 (7366)	75486 (7428)
Multiplicity	2.0 (2.0)	2.0 (2.0)	2.0 (2.09)	2.0 (2.0)
Completeness (%)	99.99 (100)	100 (100)	97.5 (100.0)	100 (99.9)
(Average I)/sigma	28.4 (6.4)	23.5 (3.1)	9.9 (4.2)	7.4 (1.7)
Wilson B-Factor	16.54	14.87	19.60	28.92
R_{merge}	0.011 (0.086)	0.019 (0.211)	0.034 (0.160)	0.082 (0.466)
CC1/2	1.000 (0.981)	0.998 (0.997)	0.998 (0.942)	0.992 (0.630)
**REFINEMENT**				
R_{work}	0.1468 (0.1787)	0.1570 (0.2130)	0.1881 (0.2575)	0.2104 (0.2802)
R_{free}	0.1626 (0.2280)	0.1758 (0.2450)	0.2309 (0.2945)	0.2320 (0.3267)
**RMSD in:**				
Bond Lengths (Å)	0.018	0.016	0.003	0.005
Bond Angles (°)	1.502	1.140	0.532	0.73
**Ramachandran:**				
Favored (%)	98.99	98.66	96.63	95.42
Allowed (%)	1.01	1.34	3.37	4.15
Outliers (%)	0	0	0	0.43
Rotamer outliers (%)	1.10	0.38	0.6	1.21
**B-Factors:**				
Average	22.79	21.69	25.45	41.37
Macromolecules	19.49	18.42	24.79	41.56
Solvent	38.69	36.64	31.58	40.01

$R_{merge} = \Sigma \mid I - \langle I \rangle \mid / \Sigma I$, where I = observed intensity, <I> = average intensity over symmetry equivalent.

RMSD, root mean square deviation.

$R_{work} = \Sigma \mid |F_o| - |F_c| \mid / \Sigma |F_o|$.

R_{free} was calculated from 5% of reflections, chosen randomly, which were omitted from the refinement process.

Statistics for the highest-resolution shell are shown in parentheses.
